# Electronic Correlations in Altermagnet MnTe in Hexagonal Crystal Structure

**DOI:** 10.3390/ma18112637

**Published:** 2025-06-04

**Authors:** Evgenii D. Chernov, Alexey V. Lukoyanov

**Affiliations:** M.N. Mikheev Institute of Metal Physics of Ural Branch of Russian Academy of Sciences, 620108 Ekaterinburg, Russia; chernov_ed@imp.uran.ru

**Keywords:** altermagnetism, electronic correlations, DFT, magnetism

## Abstract

In this article, we present the results of the first-principles study of altermagnet MnTe crystallized in the hexagonal-type crystal structure. Our theoretical calculations have been performed within density functional theory (DFT) and demonstrated that the altermagnetic phase of MnTe has the lowest total energy corresponding to the stable ground state. The calculations carried out accounting for electronic correlations in DFT+U resulted in significant changes in the electronic structure, as well as magnetic properties of altermagnet MnTe and the increased bandgap. In additional calculations with spin-orbit coupling and electronic correlations (DFT+U+SO), we showed that the bandgap is less than in the DFT+U calculations, but the electronic structure did not change noticeably. In addition, the investigated pressure effects for the compound under study revealed an insulator to metal transition under pressure for the hexagonal-type crystal structure. An experimental finding of a metallic state can be complicated by structural transitions into other phases, not considered in our study, which can occur at high pressures. Experimental measurements for MnTe above 40 GPa are required.

## 1. Introduction

In recent years, the novel phenomenon altermagnetism attracted the attention of the scientific community. Altermagnetic materials are a new class of magnetic compounds in addition to common magnetic phases such as ferromagnets, antiferromagnets and others [1,2]. Ferromagnetic materials have spin-split energy bands because of the broken time-reversal (T) symmetry. Collinear antiferromagnetic materials have spin-degenerate energy bands due to the antiparallel configuration of magnetic moments and zero net magnetization. Altermagnetic compounds combine the advantages of both ferromagnets and antiferromagnets, namely, they are characterized by compensated antiparallel magnetic order, spin splittings, electronic structure of compounds with broken time-reversal (T) symmetry, magneto-optical effects, anomalous Hall effect and tunnel magnetoresistance [3,4]. It is important to note that such properties are possible due to specific crystal and magnetic structures of altermagnetic compounds [5,6]. Altermagnetics are candidates to be used in spintronic, magnetic, and memory devices [5,7,8]. Some popular compounds among altermagnets, as well as those firstly studied are RuO_2_ [9,10], FeF_2_ [11], MnF_2_ [12], among others. Recently, altermagnets were proposed among organic compounds such as k-(BEDT-TTF)_2_Cu[N(CN)_2_]Cl [13].

Among the variety of altermagnetic materials, the hexagonal manganese telluride MnTe is of particular interest. MnTe belongs to the class of transition metal chalcogenides (TMC), which are of great interest coming because of their remarkable properties [14,15,16]. In particular, they have potential for thermoelectric devices [17]. Various works are dedicated to research of pressure on crystal structure, magnetic and other properties of TMCs such as MnS and MnSe [18,19,20]. It is known that MnS and MnSe have several crystal phases of which the rock salt (RS) phase is the most energetically preferable [21]. In recent work, it was shown that RS-MnS has a phase transition under pressure. RS-MnS transit into a orthorhombic phase B31 at 24.3 GPa till 33.6 GPa accompanied by a volume collapse of 22% [22].

Unlike MnS and MnSe compounds, MnTe has the hexagonal structure of NiAs (space group $P6_{3}/mmc$) and there is no research that demonstrates the influence of pressure to MnTe, apart from pressures to 40 GPa reporting an insulating characteristics and a distorted phase for powder [23] and polycrystalline [24] MnTe samples. Combining all of the above properties, the altermagnetic MnTe is of particular interest. Altermagnetic MnTe is known to be a p-type semiconductor with a bandgap width of 1.27–1.46 eV and a magnetic ordering below the Néel temperature T*_N_* = 307–310 K [25] with magnetic moment of 4.7–5.0 $\mu_{B}$ [26]. In theoretical calculations [5,15,27,28,29,30,31,32], the bandgap was underestimated. Recently, the existence of the altermagnetic ordering in MnTe was demonstrated by revealing an altermagnetic band splitting in the Brillouin zone, which is present even in the absence of spin-orbit coupling (SOC) [3,5,15]. Because of this, further studies of MnTe are required to understand its properties and potential applications.

In this paper, we performed a first-principles theoretical study employing DFT+U to reveal the electronic structure and magnetic properties of the altermagnet MnTe with different unit cell volumes and demonstrate an insulator-metal transition under pressure. The motivation of our work is to demonstrate the importance of taking into account electronic correlations for the reproducing of the MnTe bandgap because a usual DFT calculation cannot reproduce the experimental value of the bandgap in this insulator.

## 2. Methods and Crystal Structure

**Figure 1:**
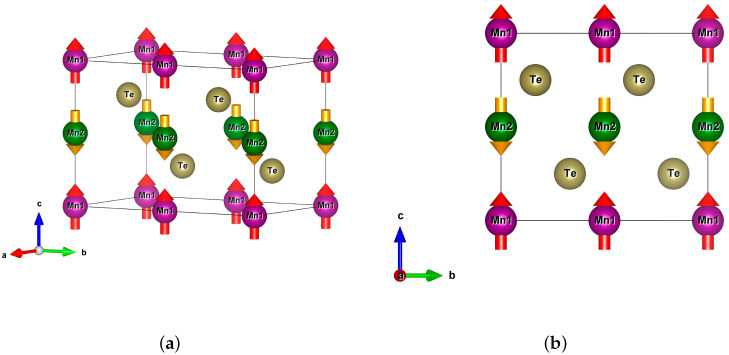
(**a**) The motif of crystal structure of MnTe. (**b**) The crystal structure of MnTe in *yz*-plane. Mn1 atoms are purple, Mn2 atoms–green, Te atoms–yellow. Arrows correspond to directions of magnetic moments.

The altermagnetic MnTe crystallizes in the hexagonal $P6_{3}/mmc$ crystal structure (space group number 194) [15]. The crystal structure is plotted in Figure 1. The manganese Mn1 and Mn2 ions correspond to different directions of magnetic moments shown as arrows. In order to compare different types of antiferromagnetic ordering in MnTe, we increased the size of the cell along the *b* direction and selected a spin ordering presented in Figure 1. Our cell contains 4 Mn and 4 Te atoms. The cell parameters are: a = 4.21 Å; b = 8.42 Å; c = 6.70 Å and α = β = 90°; γ = 120° [33]. The crystal structure of MnTe was plotted in VESTA [34]. The investigations were performed within density functional theory (DFT) methods. In this type of calculations, temperature is not explicitly present as a parameter, while it characterizes the crystal structure parameters taken. To take into account electronic correlations, the DFT+U method was used with Coulomb parameter varied from 3 to 6.9 eV, the exchange parameter equals to J = 0.86 eV [20,35]. The Hubbard U and exchange parameter are applied for the Mn 3d states. The electron-ion interaction pseudopotential was based on the projector augmented wave method and the exchange–correlation functional was chosen in the GGA-PBE form [36]. The calculations with spin-orbit coupling were performed with U = 6.9 eV and J = 0.86 eV with non-collinear antiparallel orderings of the Mn magnetic moments in the *xy*-plane. The cutoff energy was set as 520 eV and the k-grid mesh was 8 × 16 × 8 points. The calculations were performed in VASP package ver. 5.4 [37,38].

## 3. Results

**Figure 2:**
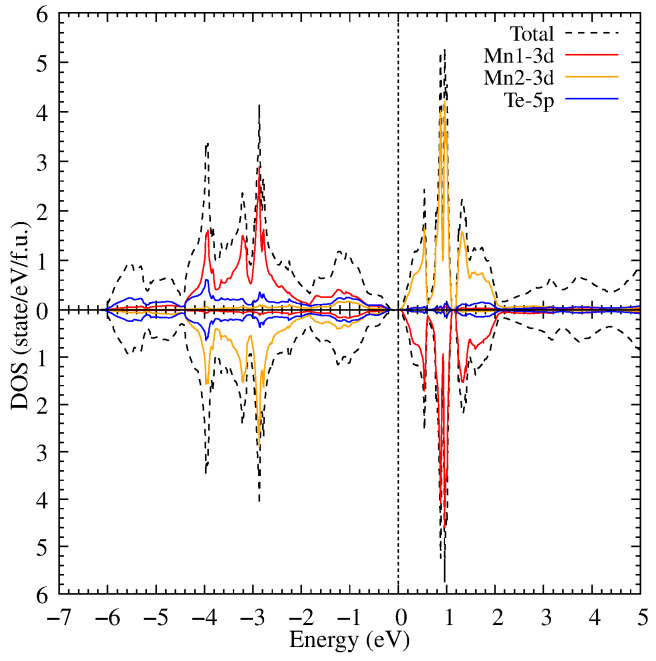
The density of electronic states of MnTe in the GGA approximation relative to the Fermi energy at zero energy (the dotted line).

First, we performed calculations with different types of antiferromagnetic orderings. A comparison of total energies showed that the altermagnetic ordering presented in Figure 1 has the lowest total energy. After this we performed a relaxation calculation of crystal structure to take into account the altermagnetic spin ordering. Figure 2 presents the electronic structure in the GGA approximation. The Mn1 ions the spin up projection is occupied, Mn2–the spin down channel is occupied. It can be observed that MnTe exhibits a semiconductor bandgap width of 0.2 eV. In the valence part of the electronic states, several 3d Mn peaks are observed at −4, −3.2 and −2.9 eV. Also, several 3d Mn peaks are placed in the conduction part of the electronic states at 0.6, 1.0 and 1.2 eV. These results are in good agreement with the previous theoretical calculations [39,40].

**Figure 3:**
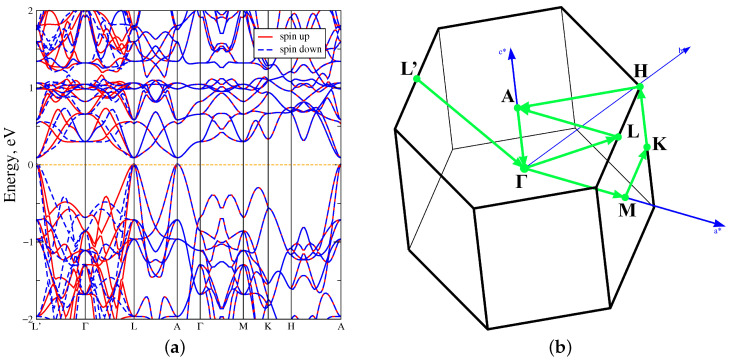
(**a**) The band structure of MnTe in the GGA approximation. (**b**) The Brillouin zone of MnTe with high-symmetry points denoted by green dots.

Figure 3a demonstrates the band structure in the L’–Γ–L–A–Γ–M–K–H–A direction of MnTe in the GGA approximation. Also the MnTe Brillouin zone with the direction of the bypass high-symmetry points is demonstrated in Figure 3b. The red lines indicate the spin up projection, blue dot lines–the spin down projection. It is seen, that bands in the spin up and down projections are matched in the direct L–A–Γ–M–K–H–A intervals, which indicates antiferromagnetic nature. But a spin splitting as a result of the broken time-reversal symmetry (T) is observed in the reverse L’–Γ–L direction. For this reason, the spin up and spin down projections are mirrored but indicate the ferromagnetic nature. All these facts confirm that MnTe exhibits altermagnetic properties.The bandgap width is equal to 0.2 eV due to the presence of states in the vicinity of the Fermi level at L’, L and A high-symmetry points. In turn, at the other high-symmetry points, such as Γ, the direct energy gap width is 1 eV. Also the bandgap width achieves 1.75 eV at the H point.

**Figure 4:**
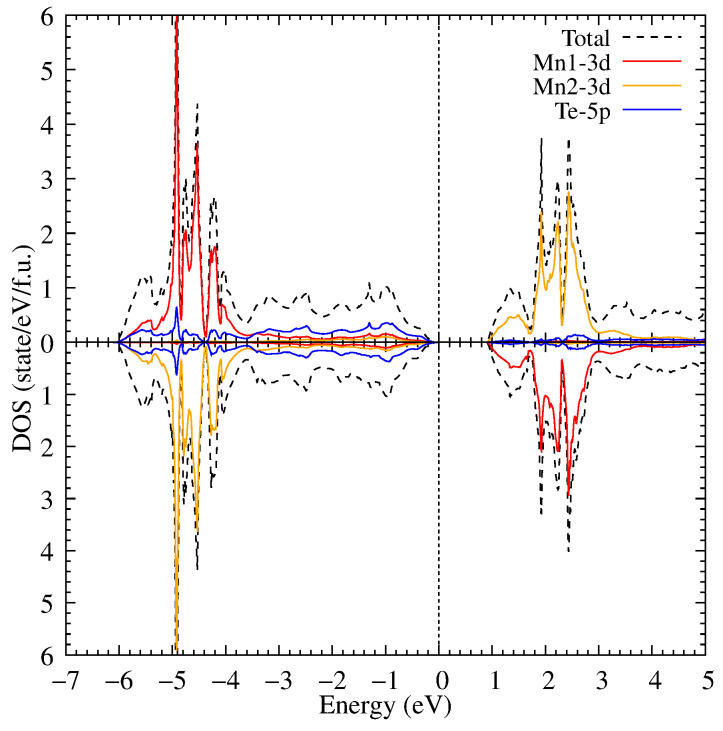
The density of electronic states of MnTe in DFT+U for U = 3 eV.

Further, electronic correlations in the Mn 3d shell with Hubbard U = 3 eV were taken into account. As a result of accounting for electronic correlations, the bandgap width increased to 0.9 eV (see Figure 4) due to the shift of the 3d Mn states in the valence part of the electronic states to the lower energies.

Now, the 3d Mn states have intense peaks at −4.1, −4.5 and −5.0 eV. In its turn, the electronic correlations also influence the 3d Mn states in the conduction band which are shifted to the higher energies. The electronic correlations have weak effect on the Te 5p states. Most Te states are not transferred; however, the Te states hybridized with the Mn ones are moved to the lower energies in the valence band.

**Figure 5:**
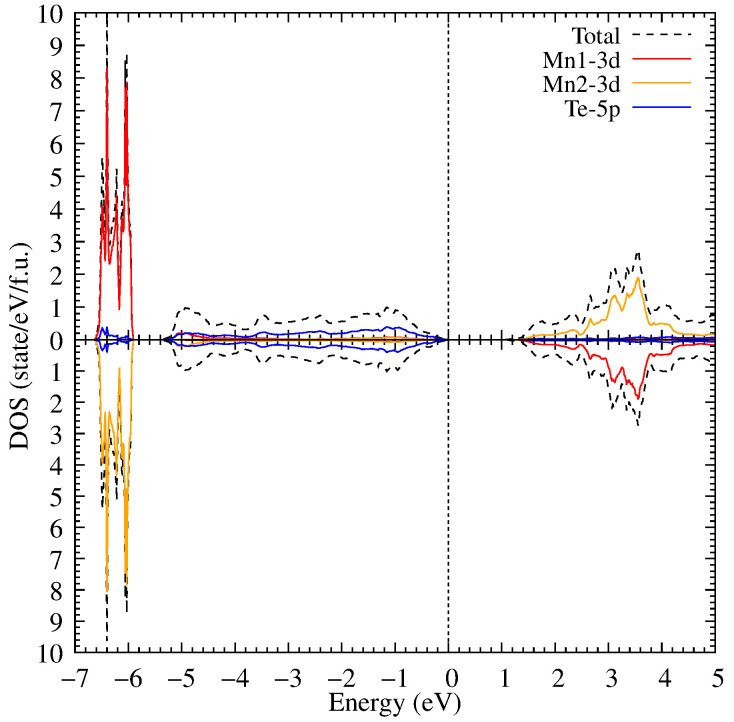
The density of electronic states of MnTe in the framework of the DFT+U method for U = 6.9 eV.

After increasing the U value up to 6.9 eV, the bandgap width is increased slightly in comparison with U = 3 eV, see Figure 5. The Mn 3d states separated from the Te 5p ones in the valence band form a gap between −5.8 and −5.2 eV. And now the Mn 3d states are located between −5.8 and −6.6 eV with the higher peak intensity than in the previous calculation. This means that the main part of the Mn 3d states in the valence band is shifted to an area of −5.8 to −6.6 eV. In addition, a small part of the Te 5p hybridized states is shifted together with the Mn 3d states. The obtained DOS is in good agreement with the experimental photoemission spectra [41] where the occupied states are found just below 0 and a pronounced peak between −7 and −6 eV. The Mn 3d of the conduction part are moved by 1 eV to the above energies. This leads to the bandgap width increase because these states are found above 1.25 eV in the conduction part; notable, the Te 5p limit the bandgap in the valence band. The position of these states is also in agreement with the experimental photoemission spectra [41] having the main contribution of the Mn states between 3 and 4 eV.

**Figure 6:**
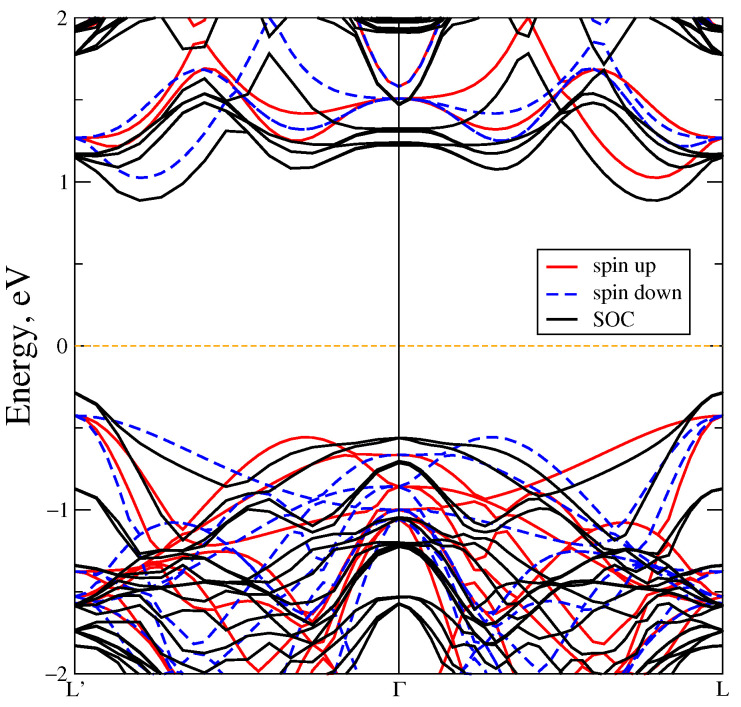
The band structure of MnTe in DFT+U for U = 6.9 eV (red and blue stripes) and with taking for account spin-orbit coupling (black stripes).

The band structure also undergoes significant changes; see Figure 6. The bandgap width in the L’–Γ–L direction increases to 1.5 eV, whereas the energy gap width is 2 eV in the Γ point. An indirect gap also exists along the L–K path.

Also, we performed calculations taking for account spin-orbit coupling together with Coulomb parameter equal to 6.9 eV, see Figure 6. Taking SOC into account does not result in noticeable changes in the states, and the bandgap decreases slightly by 0.2 eV. This effect is comparable with a bandgap change of 0.12 eV when the exchange-correlation functional LSDA is replaced by GGA-PBE in the calculations. The energy of SOC for the Mn ions is equal to −0.008 eV, and for the Te ions it is −0.189 eV. It is clear that the Te SOC energy is much higher than the Mn SOC energy.

**Figure 7:**
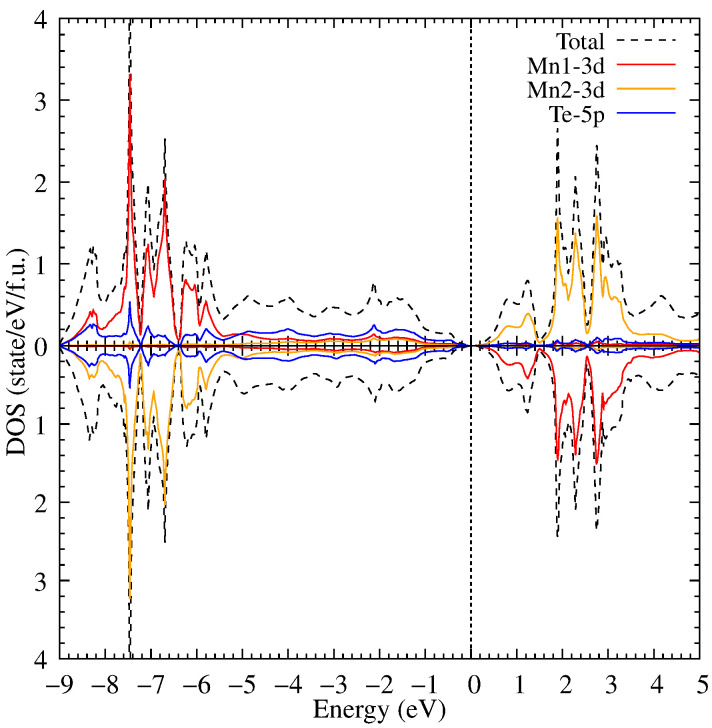
The density of electronic states of MnTe in DFT+U for U = 6.9 eV and with a volume of 70% of the volume under normal conditions.

In addition, in this work, modeling of the unit cell compression of MnTe was realized by reducing the lattice volume and keeping the ratio of the lattice parameters with the electronic correlations taken into account. The compression was considered without taking into account possible crystal structure transitions. Then the result of the calculations for a volume of 70% of the initial volume (i.e., the volume under normal conditions) is presented in Figure 7. As a result of the applied pressure, the narrow peaks of manganese are broadened. The intensity of the Mn peaks is also noticeably decreased. The bandgap is reduced to 0.4 eV due to the above changes and the shift of the Mn states. These results are in agreement with the available results that report insulating characteristics up to 40 GPa [23,24]. It might be expected that above this pressure a metallic phase can be found.

**Figure 8:**
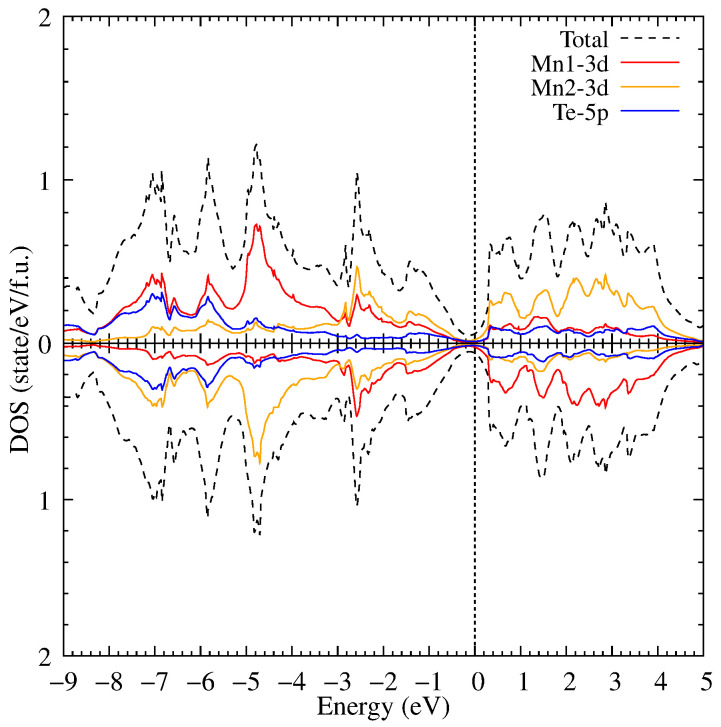
The density of electronic states of MnTe in DFT+U for U = 6.9 eV and with a volume of 50% of the initial volume under normal conditions.

**Figure 9:**
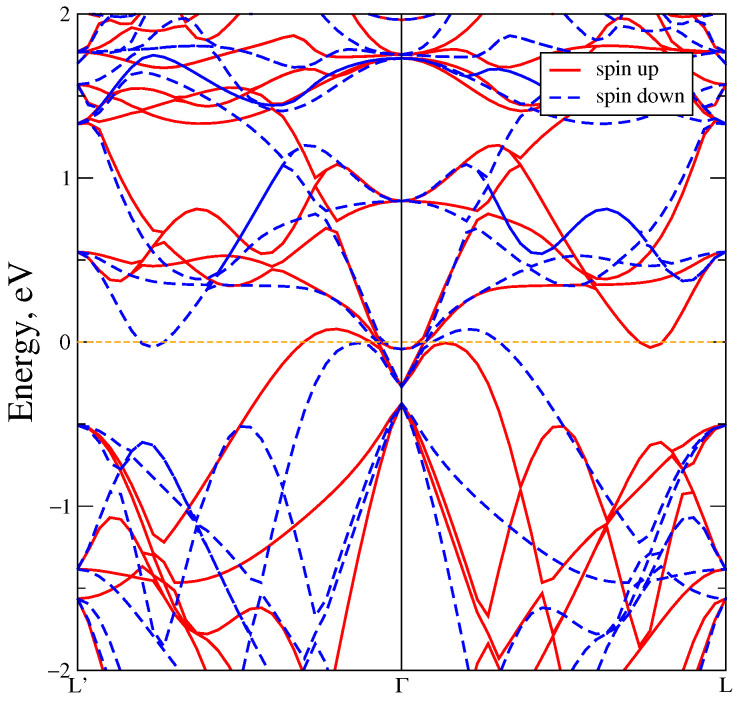
The band structure of MnTe in DFT+U at U = 6.9 eV and with a volume of 50% of the normal conditions volume.

With a further decrease of volume to 50% (see Figure 8) of the initial volume, the gap is closed and an insulator-metal transition occurs. The intensities of the Mn 3d states decrease significantly, and the Mn 3d–Te 5p states strong hybridization is observed. The band structure presented in Figure 9 overlaps at the point Γ at the Fermi level with a large altermagnetic spin splitting.

**Figure 10:**
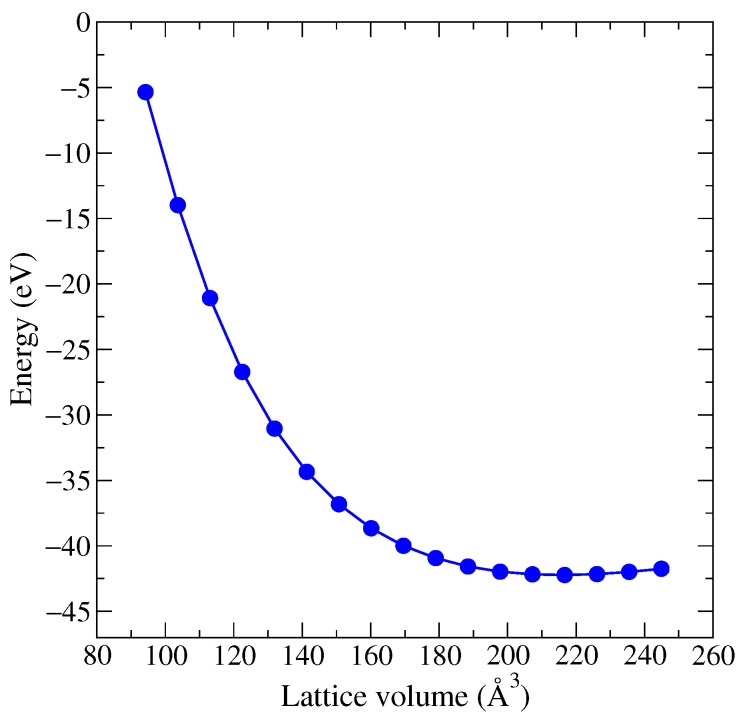
The curve of equation of states for MnTe.

During our investigation of the pressure effects on the electronic structure, as well as magnetic properties, of altermagnetic MnTe, bulk modulus B0 was also calculated. The Birch-Murnaghan equation of states was also obtained and plotted, see Figure 10. In order to compress MnTe by 15% (160.1 Å), a pressure of 27.5 GPa is required. A pressure of 47.8 GPa results in a volume reduction of 25% (141.3 Å). And a pressure of 177.6 GPa is required to compress MnTe by 50% (94.21 Å). The B0 value was calculated as 53.7 GPa and B’0 = 3.54. The bulk modulus of MnTe was previously theoretically calculated as 17.54–50.84 GPa and B’0 ranging between 3.85–5.83 for different parameters [42], which shows reasonable agreement with our calculated results.

**Figure 11:**
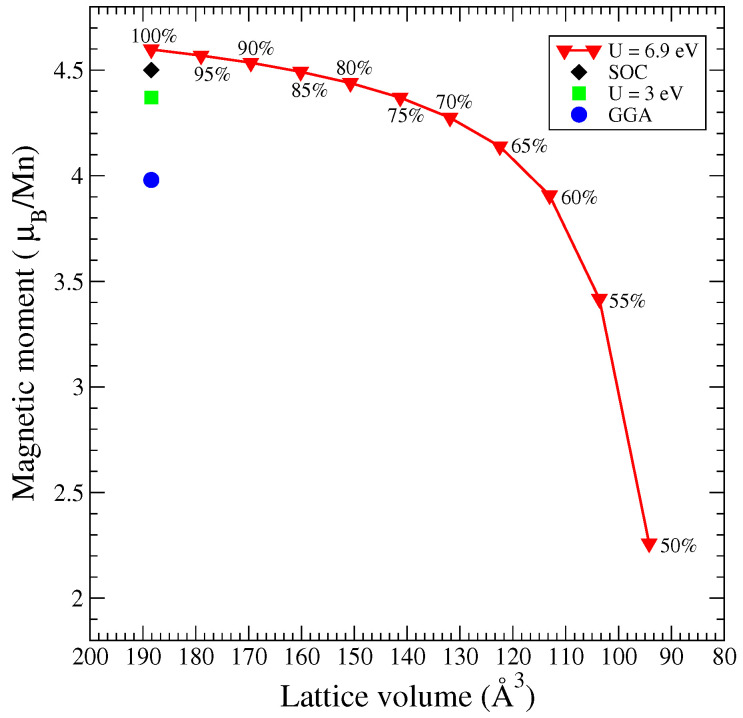
The Mn magnetic moments in MnTe depending on a calculation method and volume at U = 6.9 eV.

Because MnTe exhibits altermagnetic properties, the total magnetic moment for it is equal to zero Bohr magnetons, as the magnetic moment of the Te ions. However, the Mn magnetic moment has a high value in all our computational methods; see Figure 11. Thus, the Mn magnetic moment is equal to 3.98 $\mu_{B}$ in the GGA calculation. As the Coulomb parameter increases to 3.0 eV, the magnetic moment of manganese increases to 4.37 $\mu_{B}$. At the maximum value of the Coulomb parameter 6.9 eV [20,35], the Mn magnetic moment reaches 4.6 $\mu_{B}$, which is characteristic for the high spin state. The Mn magnetic moment in the case of a calculation with SOC is equal to 4.5 $\mu_{B}$. Furthermore, with a decrease in the volume of the MnTe cell, the Mn magnetic moment decreases. Under compression, the energy bands expand, and as a result, the magnetic moment on Mn decreases. When volume of 50% is reached (with the Mn magnetic moment of 2.26 $\mu_{B}$), a transition into a low spin state is finished.

## 4. Conclusions

We studied the electronic structure and magnetic properties of altermagnetic MnTe taking into account electronic correlations. In addition, we investigated the effects of pressure on the electronic structure and magnetic properties of the compound under study. It is shown as Coulomb parameter increases, the bandgap width is increasing to 1.25 eV. This value is close to the experimental one. Also as Coulomb parameter increases, Mn magnetic moments are growing to 4.6 $\mu_{B}$. Our calculations taking into account the spin-orbit coupling show a decrease of the bandgap to 0.68 eV in comparison with the LSDA calculations and the Mn moment to 4.5 $\mu_{B}$. We have demonstrated that it is not necessary to take SOC into account in order to reproduce the altermagnetic properties. The volume compression of the cell leads to a gradual decrease in the Mn magnetic moment with a decrease in the volume of the cell, resulting in a transition from a high-spin state to a low-spin one. When 60% of the volume under normal conditions is reached, an insulator-metal transition occurs, as a result of which the energy gap closes, and when 50% of the volume under normal conditions is reached, the magnetic moment of manganese decreases to 2.26 $\mu_{B}$, but altermagnetic spin splitting is present. Furthermore, in the course of our study, the values of the bulk modules B0 and B’0 were calculated. Our results motivate further experimental studies of altermagnetic MnTe above 40 GPa.

## Data Availability

The original contributions presented in this study are included in the article. Further inquiries can be directed to the corresponding author.

## Abbreviations

The following abbreviations are used in this manuscript:

TMC	Transition metal chalcogenides
DFT	Density functional theory
RS	Rock salt
SOC	Spin-orbit coupling
LSDA	Local spin-density approximation
GGA	Generalized gradient approximation
